# Comparing the Bayesian Unknown Change-Point Model and Simulation Modeling Analysis to Analyze Single Case Experimental Designs

**DOI:** 10.3389/fpsyg.2020.617047

**Published:** 2021-01-15

**Authors:** Prathiba Natesan Batley, Ratna Nandakumar, Jayme M. Palka, Pragya Shrestha

**Affiliations:** ^1^College of Health and Life Sciences, Brunel University London, Uxbridge, United Kingdom; ^2^School of Education, University of Delaware, Newark, DE, United States; ^3^Department of Educational Psychology, University of North Texas, Denton, TX, United States

**Keywords:** single case design, Bayesian, Markov Chain Monte Carlo Method, statistical simulation model, interrupted time series analysis, single case experimental designs

## Abstract

Recently, there has been an increased interest in developing statistical methodologies for analyzing single case experimental design (SCED) data to supplement visual analysis. Some of these are simulation-driven such as Bayesian methods because Bayesian methods can compensate for small sample sizes, which is a main challenge of SCEDs. Two simulation-driven approaches: Bayesian unknown change-point model (BUCP) and simulation modeling analysis (SMA) were compared in the present study for three real datasets that exhibit “clear” immediacy, “unclear” immediacy, and delayed effects. Although SMA estimates can be used to answer some aspects of functional relationship between the independent and the outcome variables, they cannot address immediacy or provide an effect size estimate that considers autocorrelation as required by the What Works Clearinghouse (WWC) Standards. BUCP overcomes these drawbacks of SMA. In final analysis, it is recommended that both visual and statistical analyses be conducted for a thorough analysis of SCEDs.

## Introduction

Single case experimental designs (SCEDs) investigate change within an individual or a sampling unit rather than aggregate change for a group of individuals or units. Fields of applications of SCEDs include special education, psychology, and medicine, among others (e.g., Guyatt et al., 1986; Allen et al., 2013). SCED studies are interrupted time series designs where an outcome variable is assessed repeatedly for an individual (or unit) over different phases. There is at least one baseline (phase A) and one intervention phase (phase B), with multiple observations both before and after treatment.

Single case experimental designs have traditionally relied on visual analysis of graphs from multiple phases for determining the presence and magnitude of a treatment effect. Often visual analysis reports are supplemented with reporting phase means, medians, percentages, and effect sizes such as standardized mean differences or indices based on the amount of data overlap between phases (Parker et al., 2007). Although visual analysis has definite advantages with analyzing SCED data, studies have shown that the presence of autocorrelation can confound the results of visual analysis. For instance, in data with autocorrelation, it is difficult to decompose patterns due to trends (slopes) versus patterns due to autocorrelated errors. Natesan Batley and Hedges (2020) conducted a simulation study to demonstrate the lack of accuracy of slope and autocorrelation estimates in SCEDs when both these parameters are estimated due to indeterminacy. Autocorrelation is almost impossible to detect by visual analysis alone (Kazdin, 2011; Thyer and Myers, 2011). The presence of autocorrelation increases Type-I errors (Matyas and Greenwood, 1990) and decreases interrater reliabilities (Brossart et al., 2006) in visual analysis. In fact, Jones et al. (1978) found that in data with moderate-high autocorrelations, visual analysis results were reduced to nearly chance levels. Therefore, there is increasing emphasis for more objective methodologies for analyzing SCED data and determining causal inferences. What Works Clearinghouse (WWC; Kratochwill et al., 2013), American Speech-Language-Hearing Association (2004), and Cook et al. (2014), and Council for Exceptional Children [CEC] (2014) have all developed standards for SCEDs.

Single case experimental design data analyses pose challenges for many reasons. First, the sample sizes are often not adequate to carry out traditional analyses typically used with grouped data (Shadish and Sullivan, 2011). Second, the observations are not independent often because they are repeated measurements of the same individual. Therefore, the errors of SCED observations typically exhibit autocorrelation (Huitema, 1985). Most parametric and non-parametric analyses assume independence of observations. Third, although maximum likelihood-based approaches can be used to accommodate and model autocorrelations, these approaches require larger sample sizes. Finally, most SCED data are count or proportion data (Rindskopf, 2014; Shadish et al., 2014). This further exacerbates the issues with using traditional ANOVA and regression-type analyses with SCED data.

Recently, there has been an increased interest in developing statistical methodologies that can address the problems posed by SCED data and supplement visual analysis. Examples of statistical developments for SCEDs *include* multilevel modeling (e.g., Moeyaert et al., 2013), semiparametric regression models (e.g., Shadish et al., 2014), fully Bayesian analysis (e.g., Rindskopf, 2014; Natesan and Hedges, 2017; Natesan, 2019; Natesan Batley, 2020a, b; Natesan Batley et al., 2020a, b, c), simulation-based analysis (e.g., Borckardt et al., 2008), and small sample corrections to standardized mean difference effect sizes comparable to the effect sizes estimated from conventional between-subjects designs (e.g., Hedges et al., 2012, 2013). Some of these approaches are simulation-driven because simulations can compensate for small sample sizes. The present study compares two such simulation-driven approaches: the Bayesian unknown change-point model (BUCP) and the simulation modeling analysis (SMA). Apart from both being simulation-driven Monte Carlo approaches, these two can be used to estimate intercepts, slopes, and autocorrelations of single-case design data. Readers may benefit from reading more basic material presented in Natesan (2019) and Natesan Batley et al. (2020a). These two studies present the methodology and the models in detail along with software codes.

Bayesian methods use Markov chain Monte Carlo (MCMC) procedures to estimate the model parameters. The MCMC procedure is simulation-based. The estimates from many iterations for each parameter form the posterior distribution of the parameter. Because the posterior distribution is a probability distribution, statistical inferences made from these are more straightforward to interpret than those from traditional confidence intervals (e.g., Gelman et al., 2013; Kruschke, 2013). The Bayesian unknown change-point (BUCP) model estimates effect sizes while also taking into account the autocorrelation between the observations. Bayesian effect size estimate does not require small sample correction unlike the one proposed by Hedges et al. (2012, 2013). Bayesian estimation is discussed in detail in the forthcoming sections.

Like the BUCP, SMA uses a simulation modeling procedure as the basis to counter the problem of small samples in SCEDs. The estimated parameters have associated *p*-values computed for the data. In addition, SMA also estimates the autocorrelations that occur due to repeated measurements on the same subject. However, SMA does not test SCED data according to the latest standards for establishing intervention effect in SCEDs (Kratochwill et al., 2013; Cook et al., 2014), particularly an effect size that considers autocorrelation in its computation and testing for immediacy. By the term establishing intervention effect, we mean the WWC standards on criteria for demonstrating evidence of relation between an independent variable and an outcome variable. These include documenting consistency of level, trend, and variability within each phase, immediacy of effect, and an effect size to demonstrate an intervention effect. Both SMA and BUCP are freely available and are easy to use.

In sum, there are two aims for this study. The first is to demonstrate the Bayesian methodology, which is the latest advance in simulation-driven approaches, for quantifying immediacy, and estimating the effect sizes for intercept differences that take into account autocorrelations. These are two aspects prescribed by the WWC standards required to establish a functional relationship between the independent and the outcome variables in SCEDs. The second aim is to compare Bayesian (BUCP) and non-Bayesian (SMA) methodologies in their effectiveness in assessing intervention effect in analyzing SCED data.

### Specific Advantages of Bayesian in SCEDs

Bayesian methods do not depend on asymptotic theory and work well with small samples, provided the prior distribution is appropriately specified (Gelman et al., 2013). Therefore, the Bayesian effect sizes computed from BUCP models do not need small sample corrections. In the traditional frequentist framework, the statistical estimate is a fixed value with an estimate of uncertainty known as standard error. Whereas, in Bayesian estimation, each parameter of interest has its own distribution. For example, when estimating mean and standard deviation, a posterior distribution is associated with each parameter. The posterior distribution can be summarized by its mean, median, mode, standard deviation, and credible or highest density intervals (HDI). For instance, a 95% credible interval is the interval spanning 95% of the posterior density. The values within the credible interval are more credible than the values outside the interval. Additionally, posterior distributions can be used to test regions of practical equivalence (ROPE, Kruschke, 2013) as opposed to conducting classical null hypothesis significance tests (NHST). Because posterior distributions are probability distributions, ROPE and credibile intervals (as opposed to confidence intervals) do not have replicability issues, unlike NHSTs and confidence intervals. Finally, Bayesian methodology provides modeling flexibility and can be extended to count and proportion data, which are more common than interval data in SCEDs.

## Methods

### The BUCP Methodology

In SCEDs change-point is the point when the intervention is introduced, which is the beginning of the intervention phase. This is referred to as the design change-point. In visual analysis change-point is known, and for an intervention to be significant, it is expected that the relationship between the independent variable and the outcome variable shifts in the desired direction from this point onward for the rest of the phase. In BUCP analysis, however, the entire data from all phases are treated as one sequence of points and the BUCP algorithm searches for the change-point(s) in the sequence where there is a substantial change in the relationship between the independent variable and the outcome variable. That is, the change-point(s) is estimated. For example, when there is a clear immediacy (evidence of treatment effective at the start of an intervention) the estimated change-point coincides with the design change-point; however, in case of delayed effect the estimated change-point will be some time after the start of the intervention. This approach is vastly different from the traditional visual analysis where the immediacy is determined by computing the difference in the medians of the first and last 3–5 observations of phases B and A, respectively, in a two-phase study. This way of determining immediacy is highly subjective due to: (a) there being no guideline on how to interpret the magnitude of this difference to establish immediacy; in fact, this magnitude depends on the scale of the outcome variable; (b) not taking into account the patterns of all the data points in the phases; and (c) the median thus computed ignoring the autocorrelations between observations. The BUCP analysis on the other hand, statistically establishes immediacy and quantifies the effect size while accounting for autocorrelations.

The BUCP methodology is briefly described here for estimating intercepts^1^. For details see Natesan and Hedges (2017). For pedagogical purposes, the basic two-phase design is considered. However, the logic is the same for complex designs and data types. The observed value of the outcome variable *y* is assumed to be continuous and normally distributed.

The observed value at the first time point in phase 1, (*y*_*p1*_), follows a normal distribution with mean ${\hat{y}}_{p\,1}$ and standard deviation σ_ε_ as shown in Eq. 1:

(1) $$y_{p\,1}∼n\,o\,r\,m\,\left({\hat{y}}_{p\,1},\sigma_{\varepsilon }^{2}\right)$$

The observed values in the following time points *t* are distributed as:

(2) $$y_{p\,t}|H_{p\,t-1},\Theta ∼n\,o\,r\,m\,\left({\hat{y}}_{p\,t|\left(p\,t-1\right)},\sigma_{e}^{2}\right)$$

In Eq. 2, *H*_*pt–1*_ is the past history, Θ is the vector of parameters, and σ_*e*_ is the white noise created by a combination of random error ($\sigma_{\varepsilon }^{2}$) and autocorrelation between adjacent time points (ρ). The relation between ρ (autocorrelation), σ_*e*_ (white noise), and σ_ε_ (random error) is,

(3) $$\sigma_{e}=\frac{\sigma_{\varepsilon }}{\sqrt{1-\rho^{2}}}$$

The rest of the time-series follow a linear procedure with lag-1 autocorrelated errors (e.g., Harrop and Velicer, 1985; Velicer and Molenaar, 2013). The linear regression model without the slope parameter and the serial dependency of the residual (*e*_*t*_) can be expressed, respectively as,

(4) $${\hat{y}}_{p\,t}=\beta_{0\,p}$$

(5) $$e_{p\,t}=\rho \,e_{p\,t-1}+\varepsilon$$

In Eq. 4, ${\hat{y}}_{p\,t}$ is the predicted value of the target behavior at time *t* in phase *p*; β_*0p*_ is the intercept of the linear regression model for phase *p*; *e*_*pt*_ is the error at time *t* for phase *p*; ρ is the autocorrelation coefficient; and ε is the independently distributed error. Let the time points in the baseline phase be denoted as 1, 2,…,*t*_*b*_ and in the treatment phase as *t*_*b* + 1_,…,*t*_*n*_. Then the intercept β_*0p*_ can be modeled as:

(6) $$\beta_{0\,p}=\left\{ \begin{array}{c} \beta_{01},\text{if}\,t\leq t_{b}\\ \beta_{02},\text{otherwise} \end{array}\right.$$

Immediacy is indicated when the mode of the posterior distribution of the change-point *t*_*b*_ is estimated to be the same as the design change-point coupled with small posterior standard deviation. This will be demonstrated in the forthcoming sections. The effect size is the standardized mean difference of the intercept estimates in the two phases under consideration.

An important aspect of Bayesian estimation is the use of priors to estimate the posteriors. In the BUCP program relatively uninformative priors are used so as to remain agnostic about our beliefs about the posterior estimates (Natesan Batley and Hedges, 2020). For instance, β is sampled from a normal distribution with mean drawn from another normal distribution with mean 0 and precision 0.0001 which corresponds to a standard deviation of 100. The precision for the β value is sampled from a gamma distribution with shape and rate of 1 each. The autocorrelation is drawn from a uniform distribution ranging from −1 to 1, the plausible values for autocorrelation. The change-point can take on any discrete value from 3 to –3 because at least three observations are needed per phase according to WWC standards and to discern a statistical pattern. In sum, all the priors considered are relatively uninformative, however, it is strongly recommended that more informative priors be used by researchers based on information about the study and from previous research (see Lambert et al., 2005; Gill, 2015 for some general examples, and Natesan, 2019 for a specific SCED example). This is because, for small samples prior distribution plays a large influence on the resulting estimates, especially for the scale parameter. There is a great variation in the specification of relatively uninformative priors and their use could lead to different inferences (Lambert et al., 2005). Readers may download the BUCP program for implementing this analysis and producing the plots in the present study from github^2^. Runjags (Denwood, 2016) is a software package that runs JAGS (Plummer, 2003) using (R Development Core Team, 2016), all of which will be required for running the BUCP program.

In some SCEDs, delayed effects (i.e., latency) may be expected; that is, the effectiveness of the intervention is observed at a later time point after the introduction of the intervention. For instance, a drug or a chemotherapy treatment may take time to take effect or a child with autism may take time to learn how to use an iPad as a medium of communication. In such cases, it is necessary to acknowledge that the design change-point is different from the actual change-point, that is, the point when the intervention begins to take effect. The BUCP model can systematically model a delayed effect and compute the correct effect size. This is an important distinction from traditional analysis where the delayed effect is ignored, and therefore the ensuing computation of the effect size estimate is inaccurate. Thus, by allowing the data to speak for themselves, the BUCP methodology can be used to test immediacy, latency, effect sizes, and testing for region of practical equivalence (ROPE; a Bayesian equivalent of statistical significance testing), all in a single analysis.

### Bayesian Statistical Significance in BUCP

The 95% credible interval (CI) of the posterior distribution of standardized mean difference determines the limits for 95% of the credible values for the effect size under this distribution. The rule of thumb generally used in between-subject designs (i.e., 0.2, 0.5, 0.8 indicate small, medium, and large effects, respectively) cannot be used to interpret SCED effect sizes. This is because it is not uncommon to find standardized mean difference-type effect sizes such as 3 or higher in SCEDs (Shadish and Sullivan, 2011) and an effect size of 1 does not necessarily indicate a clinically significant effect. In this study, an effect size is tentatively considered to be clinically significant if it is three or higher. That is, the lower bound of the 95% CI of the posterior effect size distribution should be three or larger. However, this value was chosen for illustrative purposes only. Researchers are encouraged to choose values more appropriate for their research. Moreover, only in the Bayesian framework the null or the research hypothesis can be “accepted” as opposed to being “not rejected” as in the classical framework (Kruschke, 2013).

### Simulation Modeling Analysis (SMA)

The SMA technique (Borckardt, 2006; Borckardt et al., 2008) was developed for analyzing particularly short streams (*n* < 30 per phase) of single-case time-series design data. SMA answers the question similar to that asked in a traditional NHST context: if there is no functional relationship between the independent and the outcome variable, what is the probability one would observe the relationship at least as large as is observed with observed data? Therefore, small *p*-values (e.g., *p* < 0.05) indicate phase effect. This program simulates several thousands of random data that have the same phase *n*-sizes and the same amount of autocorrelation as the observed data. Results from observed data are then compared to the distribution for the simulated random data to determine if the observed correlation is due to chance. The percentage of times the correlations of the simulated datasets are larger than the correlation from the observed data is an estimator for the *p*-value. Results of SMA include the estimates of autocorrelation, the mean and standard deviation values for the two phases, the *p*-value associated with the level change or phase effect, and an effect size (i.e., Pearson’s *r*). The program also tests the data for different standard slope change models. Users can modify the program available on http://www.clinicalresearcher.org/software.htm for other types of SCEDs.

The SMA program is a freeware and easy-to-use tool for SCED researchers. However, it is not without disadvantages. Although SMA is a sound statistical procedure, it assumes that the estimated parameters used to generate data streams are a reasonable representation of the data characteristics. Secondly, SMA ignores autocorrelation when computing Pearson’s correlation effect size. Therefore, it underestimates the effect size. Thirdly, most SCED data are count data or ratio data. It is unclear how SMA functions for such data (Borckardt and Nash, 2014). Fourthly, SMA does not function well for large data streams, although SCED data time-series are rarely longer than 10 data points per phase. Fifthly, it facilitates researchers to test several hypotheses of slope differences. Testing multiple hypotheses leads to an increase in experiment-wise type-I error rate. Moreover, a researcher may be tempted to simply test each hypothesis at the traditionally used 0.05 threshold value and report only those they find statistically significant. Finally, the program cannot estimate delayed effects. The focus in SMA is to measure treatment effect assuming there is a clear immediacy and not test all aspects of intervention effect as prescribed by the WWC for SCEDs (e.g., immediacy, appropriate effect size). In sum, although SMA is very useful, it is not a one-stop-shop for complete SCED analysis.

## Data

We selected three datasets from recent SCED literature that exhibited “clear” immediacy, “unclear” immediacy, or delayed effects (latency) to demonstrate the difference between the methods. Clear immediacy is exhibited if the data patterns in the two phases clearly supported the intervention change-point as the design change-point. Unclear immediacy is exhibited when the patterns within the phases show some inconsistency so that a researcher cannot clearly point out when the actual change began to take place. Delayed effects are exhibited when the actual change occurs at least one time point after the intervention was implemented. All of these grades of immediacy were determined by visual inspection. The team of four authors independently categorized the selected datasets as belonging to one of the immediacy types. There was 100% agreement in the categorization. The graphs were digitized using the digitizing software WebPlotDigitizer 3.11 (Rohatgi, 2017). Data that were coded manually were compared to the data coded by WebPlotDigitizer for each dataset to ensure the values were identical.

### Datasets’ Characteristics

#### Dataset 1

This dataset was obtained from a multiple baseline design (MBD) by Coulter and Lambert (2015) and classified as showing clear immediacy. The dependent variable was the percent of correct words read per minute in a preselected 150–200-word passage by participants with a learning disability and reading two grade levels below their same age peers. The researchers used visual analysis. Autocorrelation was not calculated; however, the researchers reported an overall (inclusive of three participants) percentage of all non-overlapping data (PAND) effect size = 97.91% and 90% CI = [0.94, 0.99]. In addition to PAND, Coulter and Lambert (2015) reported a Pearson’s *phi* value of 0.915, 90% CI = [0.84, 0.98]. BUCP and SMA estimates for all 3 participants in the MBD were also computed as an extension of the first case to MBDs.

#### Dataset 2

Dataset 2 was obtained from one of the subjects of a MBD by Macpherson et al. (2015) who examined the effects of a portable video modeling intervention (using iPad^®^) on the verbal compliments and compliment gestures of children with autism. Dataset 2 contains the number of verbal compliments given by one participant in the baseline and intervention phases. The authors of the study used a one-tailed Wilcoxon signed rank test to conclude the presence of a statistically significant difference between the observations in the baseline and intervention phases. Effect sizes and autocorrelation were not reported. The data show a possible *delayed* effect.

#### Dataset 3

The third dataset was taken from Barber et al. (2016) because it showed *unclear* immediacy. This study examined the efficacy of peer-mediated interventions (i.e., stay, play, and talk strategies) on the social communication skills of preschool children with autism. Barber et al. (2016) used SMA. A visual plot for the data suggests that it is unclear when the treatment effect started and whether there was a statistically significant treatment effect. SMA results showed no statistically significant level change at α = 0.05 level (Pearson’s *r* = 0.729; *p* = 0.06) but a statistically significant slope change (*r* = 0.867, *p* = 0.02). Autocorrelation was not reported.

## Results

**FIGURE 1:**
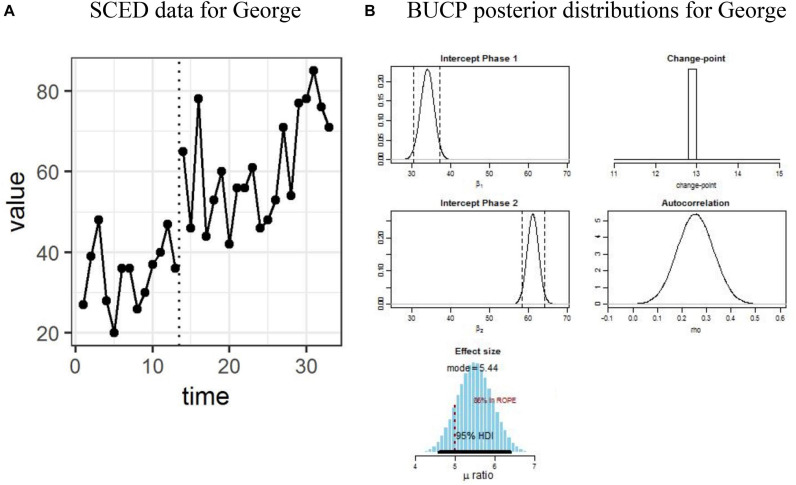
**(A,B)** Single case experimental design (SCED) raw data for George in Dataset 1 and posterior distributions obtained with Bayesian unknown change-point (BUCP).

**FIGURE 2:**
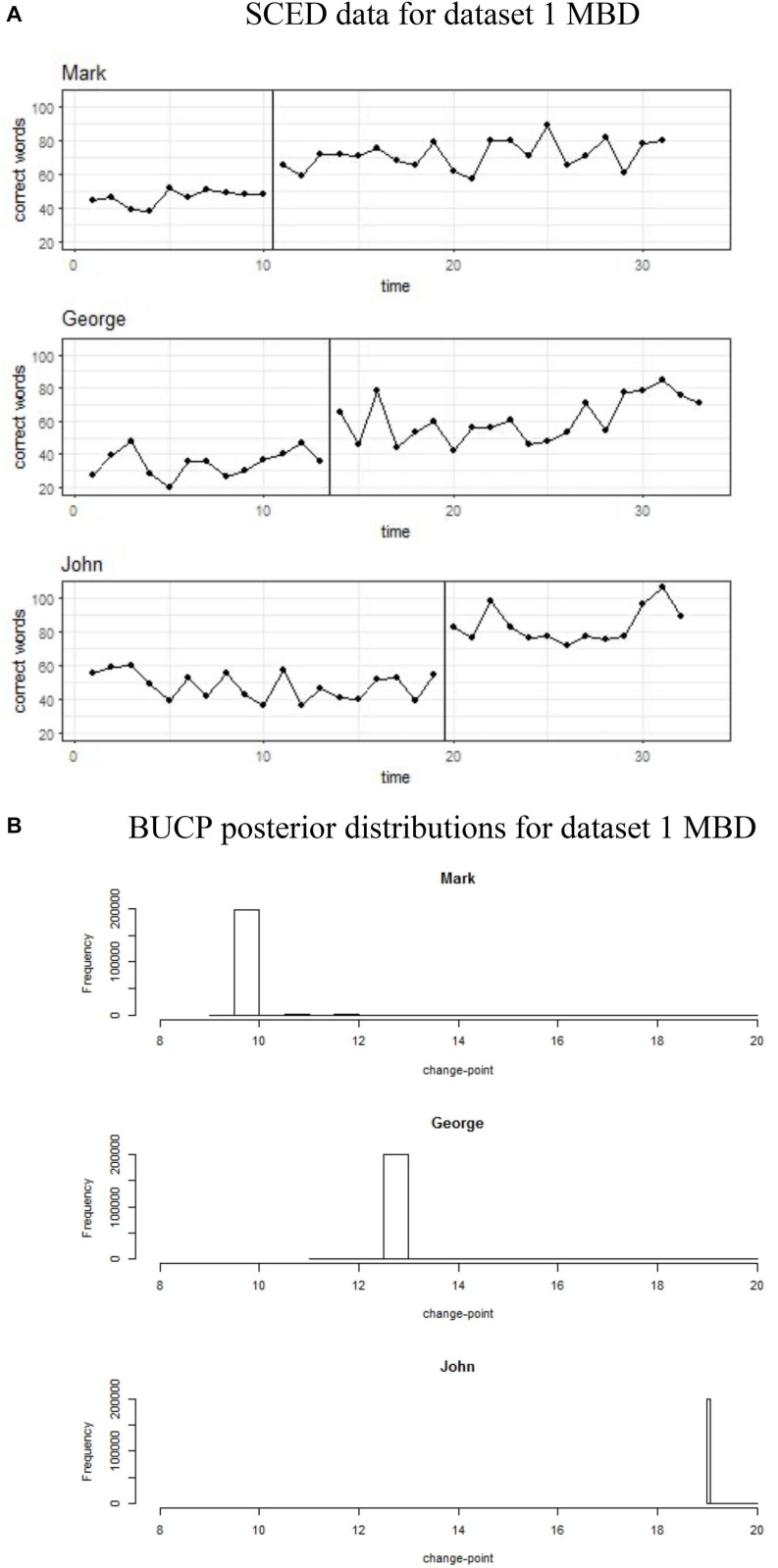
**(A,B)** Single case experimental design (SCED) raw data for MBD dataset 1 paired with the posterior distribution for change-points obtained with Bayesian unknown change-point (BUCP).

**FIGURE 3:**
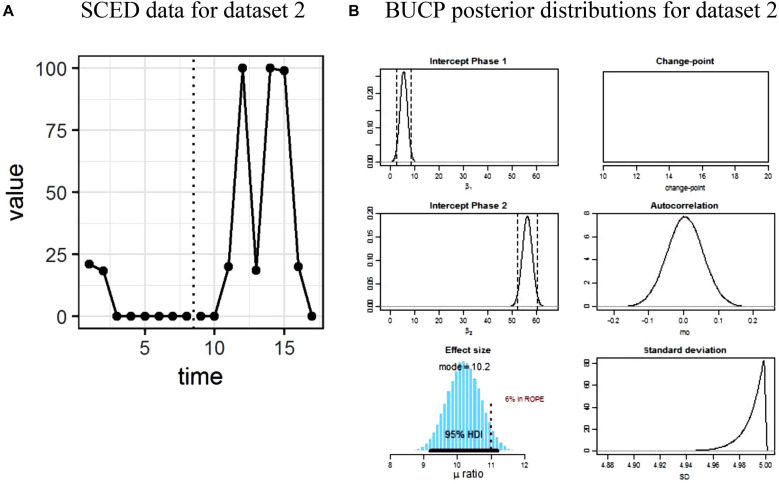
**(A,B)** Single case experimental design (SCED) raw data for dataset 2 paired with the posterior distributions obtained with Bayesian unknown change-point (BUCP).

**FIGURE 4:**
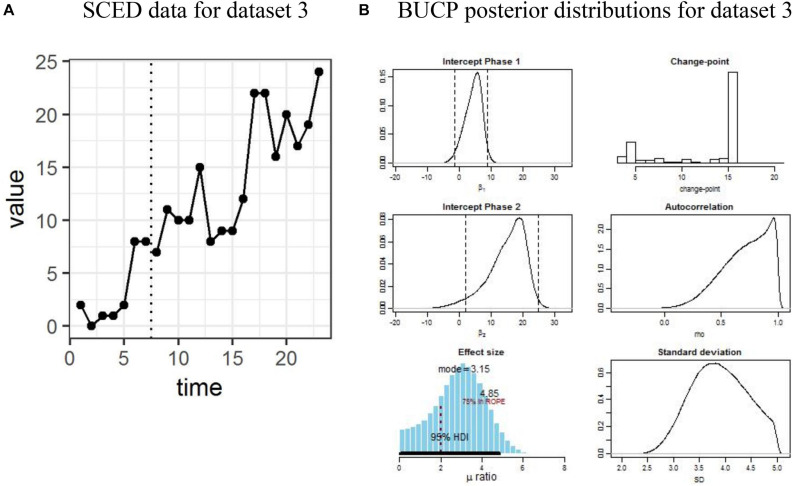
**(A,B)** Single case experimental design (SCED) raw data for dataset 3 paired with the posterior distributions obtained with Bayesian unknown change-point (BUCP).

The results for SMA and BUCP analyses for all datasets are shown in Table 1. Figures 1–4 display the line charts and posterior plots for all data sets. Each figure has two parts: Part *a* displays the line chart of the data and Part *b* displays the posterior plots. For Datasets 2 and 3, for the sake of brevity, only posterior plots for phase means and the effect size are included.

**TABLE 1:** Description of datasets and results from simulation modeling analysis (SMA) and Bayesian unknown change-point (BUCP).

Data description						SMA results		BUCP results			
Dataset		n_A_	n_B_	Mean_A (SD)_	Mean_B (SD)_	r*	*p*-value	Change point	Mean_A_ [95% HDI]	Mean_B_ [95% HDI]	ES Mean [95% HDI]
Dataset 1 (MBD)											
	George	13	20	34.62 (8.18)	61 (13.27)	0.75	0.004	13	33.93 [30.51, 37.28]	61.21 [58.30, 64.13]	5.5 [4.60, 6.38]
	Mark	10	20	46.1 (4.65)	71.1 (8.52)	0.85	0	10	46.09 [43.05, 49.15]	71.48 [69.4, 73.48]	5.2 [4.41,5.96]
	John	19	13	47.8 (8.08)	83.7 (10.59)	0.89	0.0012	19	47.27 [44.49,49.93]	83.28 [79.98, 86.63]	7.3 [6.42, 8.18]
Dataset 2		8	9	4.92 (8.55)	39.73 (43.13)	0.48	0.192	11	5.38 [2.37, 8.32]	56.22 [52.27, 60.39]	10.19 [9.21, 11.22]
Dataset 3		7	16	3.14 (3.14)	14.44 (5.48)	0.73	0.049	16	4.31 [−1.35, 8.89]	14.93 [1.92, 24.48]	2.82 [0.14, 5.37]

n, number of data points in phases A and B; HDI, high density interval or credibility interval; ES, effect size that accounts for autocorrelation; *p*, mean difference *p*-value. *r, correlation between the DV and the phase (does not account for autocorrelation).

### Clear Immediacy

#### Dataset 1: Multiple Baseline Design

The results will be described in detail for George, followed by John and Mark. The vertical dotted line in Figure 1A separates the data for the child George from the two phases. There are 13 points in Phase A and 20 points in Phase B. Visual observation of Figure 1A shows clear immediacy at the end of Phase A at time point 13. It can be seen from Table 1 that the observed means and standard deviations (SD) for Phases A and B are 34.62 (8.18) and 61.0 (13.27), respectively. The SMA analysis showed that there is a statistically significant phase effect (*p* < 0.01). That is, the means differ significantly between Phase A and Phase B. Figure 1B shows Bayesian results for the same data: posterior distribution plots of means for Phase A and Phase B, effect size, change-point, and autocorrelation. The posterior plot for the change-point shows the mode at 13, which is in agreement with a visual inspection of Figure 1A. Therefore, one can conclude that BUCP correctly estimated the change-point. Posterior distributions of means of Phases A and B are non-overlapping and narrow distributions. As can be seen from Table 1, means and 95% CIs for Phase A and Phase B posterior distributions are: 33.93 [30.51, 37.28], and 61.21 [58.30, 64.13], respectively. These estimated means are very close to the observed means, confirming our confidence in the estimation of the posterior distributions. The posterior distribution of the effect size has a mean of 5.5 with 95% CI [4.60, 6.38]. Suppose the researcher hypothesizes that an effect size greater than 3 shows a statistically significant treatment effect. Then the lower bound value of 4.6 of the 95% CI is much greater than the acceptable value of 3, indicating a large effect size and that 97.5% of credible values for the effect size are above a value of 4.6. Therefore, there is sufficient evidence to show that this effect size is statistically significant and the research hypothesis that the treatment effect is larger than 3 can be accepted.

Figure 2A shows data plots for all three children, Mark, George, and John in Dataset 1, who are part of the MBD. Figure 2B shows their respective posterior distributions for the change-point. Visual observation data for Mark shows a clear immediacy at the end of Phase A at time point 10. It can be seen from Figure 2B that the BUCP modal estimate of the change point was also 10. This shows support for immediacy. The means for Mark in Table 1 show that there was a considerable increase in the percent correct score in phase 2 (46.1 to 71.5%). The 95% credible intervals for the effect size [4.41, 5.96] do not contain zero and are above 3, indicating statistically significant improvement. These results show that, for Mark, because of the intervention, the percent correct score in Phase 2 has increased, on an average, by more than five standard deviation units (mean of the effect size posterior distribution, 5.2).

BUCP estimate of change-point for the third child, John was 19, that is, the time-point immediately after the intervention started. Thus, again, there is support for immediacy. There was a considerable increase in the percent correct score in phase 2 (47.3 to 83.3%). Credible intervals for the effect size do not contain zero, indicating improvement. Furthermore, the lower bound of the 95% credible intervals for the effect size is 6.42, much higher than the acceptable value of 3 for statistically significant difference. These results indicate that, for John, because of the intervention, the percent correct score in phase 2 has increased, on an average, by more than six SD units (mean of the effect size posterior distribution, 7.3).

### Delayed Effect

#### Dataset 2

Visual observation of Figure 3A reveals a delayed effect, with the treatment effect appearing around time point 10 rather than at 8 when the intervention started. The observed means and standard deviations for Phase A and Phase B, in Table 1, are 4.92 (8.55) and 39.73 (43.13), respectively. The SMA results which presume 8 as the change-point indicate a statistically non-significant phase effect (*p* > 0.19). The BUCP analysis estimated the change-point at 11, correctly indicating a delayed effect. Note that this posterior could not be plotted in Figure 3B because there is no variation in the change-point value in any of the iterations. Estimated means and 95% CIs for Phases A and B are 5.38 [2.37, 8.32] and 56.22 [52.27, 60.39], respectively. These means are very different from the observed means computed by considering 8 as the change-point. The posterior plot of the effect size is shown in Figure 3B with its mean at 10.19 and 95% CI [9.21, 11.22], indicating a large effect with 97.5% of credible values for the effect size above 9.21.

Suppose this delayed effect were expected as a function of the type of intervention. In such a case, because of the delayed effect, SMA has erroneously concluded that there is no treatment effect. On the other hand, the BUCP analysis, by correctly detecting the delayed effect and correctly estimating the change-point, showed a statistically significant treatment effect with a large effect size. These results show how misleading the results can be when immediacy is assumed to happen at the start of the treatment.

### Unclear Immediacy

#### Dataset 3

Visual observation of Figure 4A indicates that there is immediacy; however, it is not clear when. In other words, there is a delayed effect, but unlike Figures 1A or 3A, it is not clearly distinguishable visually when the change-point occurred. The observed means and SDs for Phase A and Phase B as shown in Table 1 are 3.14 (3.14) and 14.44 (5.48), respectively. The SMA results, which assume the time point 7 as the change-point showed a statistically significant result (*p* < 0.05). However, the BUCP analysis estimated the change-point to occur much later than when the intervention was implemented, that is, at 16 resulting in the estimated means and 95% credibility intervals as 4.31 [−1.35, 8.89] and 14.93 [1.92, 24.48], for Phases A and B, respectively. Of course, this throws some questions about the reliability and validity of the data. Specifically, are the changes in the data due to an intervention or just random fluctuations? If the researcher had sufficient information to support that the change in the data pattern is due to the intervention effect and there is a reason for the change-point to be occurring at the estimated change-point, he/she could continue with computing the effect size. The effect size posterior mean is 2.82 with 95% CI [0.14, 5.37]. The lower bound value of 0.14 for the CI is certainly below the cut-off value of 3, and more than 50% of the credible values are below the cut score of 3, clearly indicating a statistically non-significant result. Therefore, the researcher would “accept” the null that there is no statistically significant treatment effect according to the criterion set in this study.

This data set also illustrates the importance of accurately estimating the change-point. For Datasets 2 and 3, SMA results presuming the change-point to be at the time of intervention reached an erroneous conclusion while the BUCP analysis detected the delayed effect and appropriately estimated the change-point and subsequently, the posterior means. Of course, what the BUCP cannot determine is whether this delay is expected due to the nature of the intervention. This can only be determined substantively.

## Discussion and Conclusion

This study explained and discussed BUCP, a recently developed statistical methodology, for measuring effect sizes that account for autocorrelations and do not require small sample corrections for SCEDs. BUCP also investigates and quantifies immediacy in SCED studies. This study compared and contrasted the performance of BUCP with SMA, two simulation-driven approaches for analyzing SCED data. A limitation of the BUCP effect size is that it is a within subject effect size, that is, the variance used to compute it comes from the measurements of a single subject. This means that the BUCP effect size in the current study cannot be aggregated across studies in a meta-analytic context.

Determining immediacy is an important aspect of establishing intervention effect in SCEDs. However, until now, there have been no criteria as to what a meaningful difference between means/medians is to establish immediacy. Given that this difference is computed only for 3–5 data-points in each phase, testing statistical significance in the classical framework is unreasonable. This difference depends on the range of the outcome variable as well. For instance, problem behaviors may range from zero to 20 in a given time-period while performing computer mouse operation may range from zero to a few hundred. Thus, an immediacy value of 12 may indicate strong immediacy while an immediacy value of 85 may indicate weak immediacy, depending on the scale of the outcome variable. The BUCP model, on the other hand, is sensitive to patterns that show weak immediacy compared to those that show strong immediacy. This sensitivity is indicated in the shape of the posterior distribution of the change-point. If there is a clear single mode in the posterior and this mode aligns with the time point when the intervention was implemented, there is evidence to support immediacy (Natesan and Hedges, 2017).

Although visual analysts study latency, it is unclear how statisticians would deal with such delayed effects. There is also little guidance on this in existing standards. In fact, there may be no one-size-fits-all decision when it comes to delayed effect being a threat to intervention effect. When delayed effect is not considered, the effect sizes are underestimated. Therefore, examining immediacy in an objective manner is important in SCEDs. The BUCP methodology is a useful technique in this regard. It considers the entire data pattern in the two phases and estimates the change-point.

Only in the Bayesian framework the null or the research hypothesis can be “accepted” as opposed to being “not rejected” as in the classical framework (Kruschke, 2013). Since the publication of SMA in 2008, several standards for establishing intervention effect in SCEDs have been published (e.g., Kratochwill et al., 2013; Cook et al., 2014). Unlike BUCP, SMA does not model two aspects of these standards, effect sizes that account for autocorrelation and immediacy of intervention effect. For example, when there was clear immediacy, the means of the two methods were comparable. However, when there was delayed effect, only BUCP was able to identify and incorporate this delayed effect in its effect size computation. Hence, SMA is a good technique to analyze SCED data where there is clear immediacy, but falls short and provides inaccurate information about the effectiveness of an intervention when there is delayed immediacy. The BUCP analysis, on the other hand, is an effective tool in estimating the effectiveness of an intervention even in cases of delayed immediacy. In this sense, the BUCP analysis can also serve as a diagnostic tool.

Because the BUCP methodology is a Bayesian technique, it reveals a wealth of information about the possibilities of statistics of the parameters in the form of posterior distributions. In traditional analysis, mean or median are considered acceptable values to evaluate the outcome variable. However, posterior distributions are obtained based on repeated Monte Carlo simulations of a combination of the prior and the likelihood (data). Depending upon the shape of this distribution and the contextual information such as the sample size, one can examine the mean or the median of this distribution, in combination with credibility intervals of the desired length. The present study also demonstrated how regions of practical equivalence could be built around a hypothesized value to test statistical significance in the Bayesian framework. In addition, BUCP offers more modeling flexibility over SMA by being able to incorporate the scale of the data.

This study has illustrated and highlighted the strengths of the performance of BUCP compared to SMA on a limited set of data. However, a more extensive simulation study that compares the performance of the two models in a more systematic manner would provide a more complete comparison of the two approaches. The BUCP model is not without its drawbacks. Apart from the long computing time (about 47 s on average per analysis) and the learning curve, even though BUCP is highly sensitive to data patterns, a unimodal clear change-point estimate is not conclusive evidence of immediacy. Therefore, visual inspection of the data must always accompany interpreting statistical estimates in SCED data analysis. The two aspects of data analysis, visual and statistical, together can evaluate the causal validity of SCED findings via transparent, objective and replicable procedures.

## Data Availability Statement

The original contributions presented in the study are included in the article/supplementary material, further inquiries can be directed to the corresponding author.

## Ethics Statement

Ethical review and approval was not required for the study on human participants in accordance with the Local Legislation and Institutional Requirements. The patients/participants provided their written informed consent to participate in this study.

## Author Contributions

PN wrote the background, model, results and implications, and ran the Bayesian analysis. RN conceptualized the study and contributed to all sections of the manuscript in addition to running the SMA analysis. JP and PS conducted literature search and coded the data. All authors contributed to the article and approved the submitted version.

## Conflict of Interest

The authors declare that the research was conducted in the absence of any commercial or financial relationships that could be construed as a potential conflict of interest.

## Footnotes

1 Only estimation of intercepts (instead of both intercepts and trends) is considered because Natesan Batley and Hedges (2020) recommend to not estimate intercept, slope and autocorrelations all in the same SCED analysis.

2 https://github.com/prathiba-stat/BUCP
